# The prognostic significance of interferon-stimulated gene 15 (ISG15) in invasive breast cancer

**DOI:** 10.1007/s10549-020-05955-1

**Published:** 2020-10-19

**Authors:** Yousif A. Kariri, Mansour Alsaleem, Chitra Joseph, Sami Alsaeed, Abrar Aljohani, Sho Shiino, Omar J. Mohammed, Michael S. Toss, Andrew R. Green, Emad A. Rakha

**Affiliations:** 1 Division of Cancer and Stem Cells, School of Medicine, University of Nottingham Biodiscovery Institute, University Park, Nottingham, NG7 2RD UK; 2 Department of Laboratory Medical Science, Faculty of Applied Medical Science, Shaqra University, Shaqra, Saudi Arabia; 3 Department of Histopathology, Division of Cancer and Stem Cells, School of Medicine, The University of Nottingham and Nottingham University Hospitals NHS Trust, Nottingham City Hospital, Nottingham, NG5 1PB UK

**Keywords:** Interferon-stimulated gene 15, ISG15, Breast cancer, Progression, Prognosis

## Abstract

**Background:**

Lymphovascular invasion (LVI) is a prognostic factor in early-stage invasive breast cancer (BC). Through bioinformatics, data analyses of multiple BC cohorts revealed the positive association between interferon-stimulated gene 15 (*ISG15*) LVI status. Thus, we explored the prognostic significance of ISG15 in BC.

**Methods:**

The prognostic significance of *ISG15* mRNA was assessed in METABRIC (*n* = 1980), TCGA (*n* = 854) and Kaplan–Meier Plotter (*n* = 3951). ISG15 protein was evaluated using immunohistochemistry (*n* = 859) in early-stage invasive BC patients with long-term follow-up. The associations between ISG15 expression and clinicopathological features, expression of immune cell markers and patient outcome data were evaluated.

**Results:**

High mRNA and protein ISG15 expression were associated with LVI, higher histological grade, larger tumour size, hormonal receptor negativity, HER2 positivity, p53 and Ki67. High ISG15 protein expression was associated with HER2-enriched BC subtypes and immune markers (CD8, FOXP3 and CD68). High *ISG15* mRNA and ISG15 expressions were associated with poor patient outcome. Cox proportional multivariate analysis revealed that the elevated ISG15 expression was an independent prognostic factor of shorter BC-specific survival.

**Conclusion:**

This study provides evidence for the role of ISG15 in LVI development and BC prognosis. Further functional studies in BC are warranted to evaluate the therapeutic potential of ISG15.

**Electronic supplementary material:**

The online version of this article (10.1007/s10549-020-05955-1) contains supplementary material, which is available to authorised users.

## Introduction

Although the progression of breast cancer (BC) involves a complicated multi-step process, migration is one of the principal steps that is responsible for tumour progression from the in situ to the invasive stage, stromal and lymphovascular invasion (LVI) and development of metastasis. As the outcome of cancer is largely determined by its ability to produce distance metastasis, understanding the mechanisms underlying BC metastasis and investigations of LVI is warranted. LVI is a strong prognostic factor in BC, particularly in the early-stage disease and is associated with cancer-related mortality [1–4]. However, identifying the molecular mechanisms underlying LVI and its driver genes that can be targeted to prevent or reduce metastasis remains a challenge [1].

A previous study, reported by our team, has elucidated the mechanistic association between gene expression and LVI positivity [5]. The 99 deferentially expressed genes (DEGs) that showed association with LVI exhibited 42 significantly upregulated genes and 57 significantly downregulated genes based on weighted average differences (WAD) [5, 6]. Furthermore, when gene set enrichment analysis (GSEA) [7] was applied to the molecular panel previously identified by WAD approach, cell migration was the top of the main regulators of the gene panel.

Interferon-stimulated gene 15 (*ISG15*) encodes ISG15 (ubiquitin-like protein) which is highly expressed in almost all tumours and is stimulated by type I interferons. As a consequence of immune cell infiltration into the tumour stroma and because these cells are the main source of interferon α and β, this may lead to increased ISG15 expression in tumour cells [8]. Moreover, ISG15 is detected as a conjugated protein that links to multiple target proteins in what is called ISGylation process, but it can also be found in free or unconjugated status. In ISGylation, the ISG15 protein attaches and modifies a target protein in a similar way to the ubiquitylation process [9]. ISGylation plays a key role in the inhibition of protein translation either via inhibiting eIF2α by ISGylation of dsRNA-dependent protein kinase [10] or via enhancing the cap-structure-binding activity of the ISGylated translational suppressor 4EHP [11]. ISG15 has been reported to play a significant role in tumour microenvironment via enhancing T cells, B cells and epithelial cell lines cytokines [12]. A previous study has suggested that the secretion of ISG15 has an influence on T and NK cells to prompt IFNγ production and may have a significant role in the innate immunity [13]. Moreover, the extracellular ISG15 acts as an immune adjuvant that promotes antigen-specific CD8 + T cell tumour immunity [14]. In ovarian cancer, ISG15 can stimulate CD8 + T cell proliferation by activating NK cells, which enhance the cytokines production such as IL2 and IFNγ [15]. ISG15 is also highly correlated with the expression of the macrophage marker CD68 and it appears to mediate the cellular expression of multiple cytokines in macrophage through the regulation of p38 phosphorylation [16].

High ISG15 has been shown to be accompanied by certain oncogenic proteins facilitating tumour oncogenesis by inhibiting cells that control apoptosis in both primary tumour (with high *α* and *β* interferons) and during metastasis (when the interferons decrease) [8]. However, its role in LVI and cancer metastasis remains unclear. A previous study that has investigated the role of ISG15 in tumour progression and invasion suggested that high expression of ISG15 enhances the cancer cell migration invasion and metastasis [9]. ISG15 alters multiple proteins, including focal adhesion protein, action binding or modifying proteins to improve their function or increase their stability to facilitate the cancer cell migration, such as binding with Rac1 in oral squamous cell carcinoma [17]. The upregulation of *ISG15* was identified as a critical gene strongly associated with BC progression and metastasis via modulating the cell architecture and enhancing the cancer cell motility [9]. Moreover, BC cell lines showed significantly elevated expression of ISG15 compared with normal cell lines, and the knockdown of ISG15 expression was reduced ZR-75-1 cell motility in cell migration assay compared to the wild type [18]. However, the biological and prognostic value of ISG15 expression in BC remains to be defined.

In our previous studies aiming to identify the driver of LVI in BC [1, 5], ISG15 was in the differentially identified genes associated with LVI. This study aimed to validate the prognostic significance of ISG15 defined by our gene signature [5] and evaluate its association with well-established prognostic variables including LVI, relevant biomarkers related to ISG15 function/expression and patient outcome.

## Materials and methods

### Study cohorts

*ISG15* mRNA expression was assessed using the Molecular Taxonomy of Breast Cancer International Consortium (METABRIC) datasets (*n* = 1980) [19] and The Cancer Genome Atlas (TCGA) (*n* = 854) [20]. The Illumina Human HT-12 v3 platforms (Illumina, Inc., San Diego, USA) were used in the METABRIC to analyse/evaluate mRNA extracted from primary tumour samples. In TCGA, RNASeqV2 data and clinicopathological information provided by cBioPortal website were used [21, 22]. The cut-off point was determined using the median for both METABRIC cohort (9.5) and TCGA cohort (1007) to categorise into high and low subgroups. For further validation of the prognostic significance of *ISG15* in BC, an online analytical module the Kaplan–Meier Plotter (*n* = 3951) [23] was employed.

ISG15 protein expression was evaluated using a well-characterised cohort of BC (*n* = 667) collected from patients presented to Nottingham City Hospital, NHS Trust between 1989 and 1998 as previously described [24]. Patients were classified for management purposes into clinically relevant groups based on the Nottingham Prognostic Index (NPI) and Oestrogen Receptor (ER) status as previously described [2]. Patients were divided into two subgroups according to their NPI level; patients with NPI ≤ 3.4 received no adjuvant therapy, whereas patients who had NPI > 3.4 received chemotherapy if ER status negative and received tamoxifen as a hormonal therapy if ER status was positive. Classical cyclophosphamide, methotrexate and 5-flurouracil (CMF) were used as a therapy for patients who lacked ER expression and were eligible to receive chemotherapy. This was in accordance with the local BC management protocol during the period of the study patients’ presentation. Patients in this study did not receive neoadjuvant therapy or anti-human epidermal growth factor receptor 2 (HER2)-targeted therapy.

Data on ER, progesterone receptor (PR), Ki67 expression and HER2 status were available as previously published [25, 26]. ER and PR cut-off points were previously defined as ≥ 1% [26–28]. Ki67 cut-off was previously defined as ≥ 10 [29]. Information on the clinical history, clinicopathological variables, outcome and therapy was collected from patients’ clinical notes. Outcome data included the time of development of BC and BC-specific survival (BCSS) where the latter is defined as the time in months from the date of primary surgery to the time of the patient’s death due to BC.

For further understanding the protein interactions of high ISG15, available protein data from our cohort on tumour prognostic markers (P53 and Ki67), epidermal growth factor receptor (EGFR), EMT-related marker (E-cadherin (CDH1)), stem cell marker (CD44) and stromal T infiltrative lymphocyte markers (TILs) (CD8, FOXP3 and CD68) were included in this study as per previous publications [29–35] (Supplementary Table 1).

### Immunohistochemistry (IHC)

ISG15 protein expression was evaluated using immunohistochemistry preceded by validation of the ISG15 antibody specificity using Western blot [rabbit polyclonal antibody (ab227541); Abcam Company, UK] cell lysates of MCF7, SKBR3, MB-MDA-231 and MB-MDA-468 (obtained from the American Type Culture Collection; Rockville, MD, USA). The rabbit anti-ISG15 antibody (1/800) was incubated overnight at 4 °C and after being exposed to an appropriate secondary antibody, a single band at approximately 42 KDa was detected using fluorescent secondary antibodies at (1:15,000) (IR Dye 800CW donkey anti-rabbit and 680RD donkey anti-mouse, LI-COR Biosciences, UK). The mouse anti-β-actin antibody (A5441, Sigma-Aldrich; Clone AC-15; Sigma, UK) at 1:5000 was used as a house-keeping protein and showed a band at approximately 42 KDa (Supplementary Fig. 1A and B). Moreover, as recommended by the manufacturer, HELA cell lysate was used as the positive control and the specificity of the antibody was validated with a single specific band at the predicted molecular weight (~ 42KDa) (Supplementary Fig. 1C).

To assess the distribution of ISG15 expression within the BC tissue, 14 full-face sections of BC cases, representative of various molecular subtypes and tumour grade, were stained prior to TMA staining and evaluation. We have observed that ISG15 was homogenously distributed throughout these tissue sections, which indicate suitability of TMA to assess expression of ISG15 in breast cancer epithelial tumour cells. TMA Grand Master® (3D HISTECH®, Budapest, Hungary) was used to array the tumour samples into TMAs as previously described [36]. Heat-induced citrate antigen retrieval (pH 6.0) was utilised and samples were incubated with the ISG15 antibody (1:500) at room temperature for 1 h. Finally, ISG15 immunoreactivity was detected using the Novolink Max Polymer Detection kit (Leica, Newcastle, UK) and following the manufacturer’s instructions. Normal kidney tissue was used as a positive tissue control (Supplementary Fig. 1D).

### Scoring

Nanozoomer scanner (Hamamatsu Photonics, Welwyn Garden City, UK) was used to scan the stained sections with high-resolution digital images at × 20 magnification. Modified H-score was manually used to evaluate the ISG15 cytoplasmic immunoreactivity by calculating the staining intensity and percentage of positivity. The proportion of tumour cells (0–100) was multiplied by the staining intensity (0–3) and the final scores were obtained, giving a range of 0–300 [37]. Some TMA cores were regarded as non-informative if they contain less than 15% tumour cells or when they are folded cores. A blind double scoring was performed by two researchers (YK and SA) to evaluate the interobserver concordance. Intraclass correlation coefficient (ICC) concordance showed good reliability between both observers (0.85) and discordant cases were rescored by both observers to reach a final agreement. To categorise high and low subgroups, the median was used to generate cut-off for ISG15 protein (35-H-score) expression levels.

### Statistical analysis

Statistical analysis was accomplished by using SPSS (IBM SPSS Statistics, Version 24.0). Pearson correlation test was used to assess the association between *ISG15* mRNA expression and the expression of a set of genes correlated with epithelial–mesenchymal transition (EMT) [38] and cancer cell migration [24] (*CDH1 (E-cadherin), CDH2 (N-cadherin),* Transforming Growth Factor Beta 1 *(TGFB1),* Twist Family BHLH Transcription Factor 2 *(TWIST2),* Twist Family BHLH Transcription Factor 2 *(TWIST1),* Zinc Finger E-box Binding Homeobox2 *(ZEB2),* Zinc Finger E-box Binding Homeobox1 *(ZEB1),* Snail family Transcriptional Repressor 2 *(SLUG), SNAIL,* Nuclear Factor Kappa B Subunit 1 *(NFKB1),* Lethal Giant Larvae Homolog 2 *(LLGL2),* Glycogen synthase Kinase 3 Beta *(GSK3B),* Crumbs Cell Polarity Complex Component 1 *(CRUMBS) and* Catenin Beta 1 *(CTNNB1).* The association between *ISG15* mRNA expression and the expression of Matrix Metalloproteinase (MMPs) genes in the METABRIC cohort was also analysed*.* Chi-square test was used to evaluate the correlation between the clinicopathological features and ISG15 protein expression where data are available. The prognostic significance of ISG15 expression was determined via Kaplan–Meier survival curves using log-rank test. Multivariate survival analysis was assessed by using Cox proportional hazard method. Both ISG15 mRNA/protein expressions were abnormally distributed and therefore were dichotomised by median cut-off value. The statistical significance of clinicopathological factors and survival was defined by *p* value < 0.05 (two-tailed). This work was performed according to REMARK guidelines or tumour prognostic study [39] and approved ethically by the North West–Greater Manchester Central Research Ethics Committee under the title: Nottingham Health Science Biobank (NHSB), reference number 15/NW/0685.

### Bioinformatics investigation and pathway analysis

The molecular biology of *ISG15* mRNA expression was investigated at the transcriptomic level using a subset of METABRIC and TCGA cohorts [19]. Regardless of LVI status, the restriction was based on the PAM50 molecular classification using only HER2-enriched and luminal B cases as our proteomic and transcriptomic revealed a strong association of *ISG15* expression with LVI and patient outcome in these two subtypes*.* Nevertheless, the differential gene expression (DGE) analysis was performed using the Robina implementation of Edge-R statistical tool [40]. The dichotomisation of cases into high versus low groups relied on the median of *ISG15* mRNA expression in both cohorts. Henceforth, high expression of *ISG15* mRNA in the METABRIC cohort is displayed in 364/728 cases (50.0%), whereas in the TCGA cohort, *ISG15* mRNA high expression was observed in 137/275 cases (49.8%). DGE was performed in two categories: (A) cases harbouring high *ISG15* mRNA expression against cases harbouring low *ISG15* mRNA expression within the METABRIC cohort and (B) cases harbouring high *ISG15* expression against cases harbouring low *ISG15* mRNA expression within the TCGA cohort.. Common genes that were found to drive the *ISG15* mRNA expression in both the METABRIC and TCGA cohorts were identified using the Venny 2.0 online tool [41].

To explore targetable, the online public available web-based *Gene ontology enrichment analysis and visualisation tool* (GORILLA) [42] gene set analysis tool was used to identify differentially regulated canonical pathways. This pathway analysis calculated the significantly enriched pathways for the genes common to *ISG15* mRNA overexpression in both METABRIC and TCGA cohorts based on genes differentially expressed at the *p* < 0.05 level and generated by Robina analysis, including only unbiased hits with a significant enrichment score [42].

## Results

### *ISG15* mRNA expression and association with clinicopathological parameters and outcome

In both METABRIC and TCGA cohorts, high *ISG15* mRNA expression was correlated with LVI and other variables of poor prognosis, including high tumour grade, hormone receptor negativity [ER and PR], HER2 positivity and expression of EGFR and stem cell CD44) marker (Table 1). High ISG15 expression was also associated with larger tumour size and positive nodal status in the METABRIC cohort (Table 1). Moreover, when we distributed the mRNA expression of *ISG15* according to histological tumour subtypes, high mRNA expression of *ISG15* was significantly associated with ductal no special type (NST) BC compared to lobular BC subtype in the METABRIC cohort (*p* < 0.001), but not in the TCGA cohort (*p* = 0.710) (Table 1).

**Table 1:** Association of *ISG15* mRNA expression with clinicopathological characteristics in the METABRIC (n = 1980) and TCGA (n = 895) datasets

Parameters	METABRIC cohort			TCGA cohort		
	Low *ISG15*	High *ISG15*	*p* value	Low *ISG15*	High *ISG15*	*p* value
	*N* (%)	*N* (%)		*N* (%)	*N* (%)	
Tumour size						
≤ 2.0 cm	454 (53)	405 (47)	**0.003**	131 (31)	108 (45)	0.087
> 2.0 cm	526 (48)	575 (52)		297 (48)	318 (52)	
Nodal status						
Negative	543 (53)	492 (47)	**0.018**	216 (51)	207 (49)	0.606
Positive	492 (52)	496 (53)		210 (49)	216 (51)	
Histological grade						
Grade 1 and 2	551 (59)	390 (41)	** < 0.001**	268 (58)	196 (42)	** < 0.001**
Grade 3	389 (41)	562 (59)		141 (55)	211 (60)	
Tumour histological subtypes						
Ductal NST	705 (46)	839 (54)	** < 0.001**	212 (48)	227 (52)	0.710
Lobular	105 (710	42 (29)		69 (52)	63 (48)	
Medullary-like	20 (62)	12 (38)		15 (58)	11 (42)	
Special type	139 (69)	64 (31)		14 (52)	13 (48)	
Lymphovascular invasion						
Negative	482(52)	448(48)	**0.037**	258 (46)	301 (54)	**0.002**
Positive	295(47)	340(53)		169 (57)	126 (43)	
Oestrogen receptor						
Negative	207 (44)	267 (56)	**0.002**	74 (40)	111 (60)	**0.003**
Positive	783 (52)	723 (48)		335 (52)	304 (48)	
Progesterone receptor						
Negative	416 (44)	524 (56)	** < 0.001**	150 (44)	152 (56)	**0.029**
Positive	574 (55)	466 (45)		285 (52)	261 (48)	
Human epidermal growth factor receptor 2						
Negative	883 (51)	850 (49)	**0.025**	298 (53)	296 (47)	**0.013**
Positive	107 (43)	140 (57)		54 (41)	79 (59)	
Epithelial growth factor receptor						
Negative	537 (54)	453 (46)	** < 0.001**	245 (57)	182(43)	** < 0.001**
Positive	453 (46)	537 (54)		183 (43)	244 (57)	
CD44						
Negative	542 (55)	448 (45)	** < 0.001**	249 (58)	117 (42)	** < 0.001**
Positive	448 (45)	542 (55)		178 (42)	249 (58)	

Significant correlations are in bold

High *ISG15* mRNA expression was positively correlated with the expression of EMT-related markers *LLGL2* and *CTNNB1* (Table 2) and with multiple MMPs biomarkers including *MMP9, MMP11, MMP13, MMP21 and MMP28* (Table 2).

**Table 2:** Correlation of high *ISG15* mRNA expression with mRNA expression of EMT- and MMPs-related genes

Gene names	METABRIC cohort		TCGA cohort	
	Correlation value	*p* value	Correlation value	*p* value
*EMT-related genes*				
*CDH1*	− 0.034	0.127	0.032	0.353
*CDH2*	0.010	0.650	0.004	0.912
*TGFB1*	0.041	0.068	0.065	0.056
*TWIST2*	− 0.152	** < 0.001**	0.033	0.329
*TWIST1*	− 0.042	0.061	0.014	0.688
*ZEB2*	− 0.103	** < 0.001**	− 0.038	0.268
*ZEB1*	− 0.168	** < 0.001**	− 0.044	0.197
*SLUG*	− 0.142	** < 0.001**	− 0.002	0.956
*SNAIL*	0.057	**0.012**	0.011	0.738
*NFKB1*	− 0.107	** < 0.001**	− 0.005	0.889
*LLGL2*	0.102	** < 0.001**	0.068	**0.047**
*GSK3B*	0.211	** < 0.001**	− 0.042	0.216
*CRUMBS*	− 0.018	0.422	− 0.012	0.727
*CTNNB1*	− 0.165	** < 0.001**	− 0.010	**0.040**
*MMPs-related genes*				
*MMP9*	0.218	** < 0.001**	0.030	**0.035**
*MMP11*	0.152	** < 0.001**	0.116	**0.001**
*MMP13*	0.055	**0.004**	0.023	**0.023**
*MMP14*	0.065	**0.015**	0.008	0.854
*MMP15*	0.096	** < 0.001**	0.044	0.198
*MMP20*	0.050	**0.025**	0.016	0.639
*MMP21*	0.045	**0.047**	0.097	**0.005**
*MMP25*	0.076	**0.003**	0.011	0.746
*MMP28*	0.248	** < 0.001**	0.099	**0.004**

Significant correlations are in bold

**Fig. 1:**
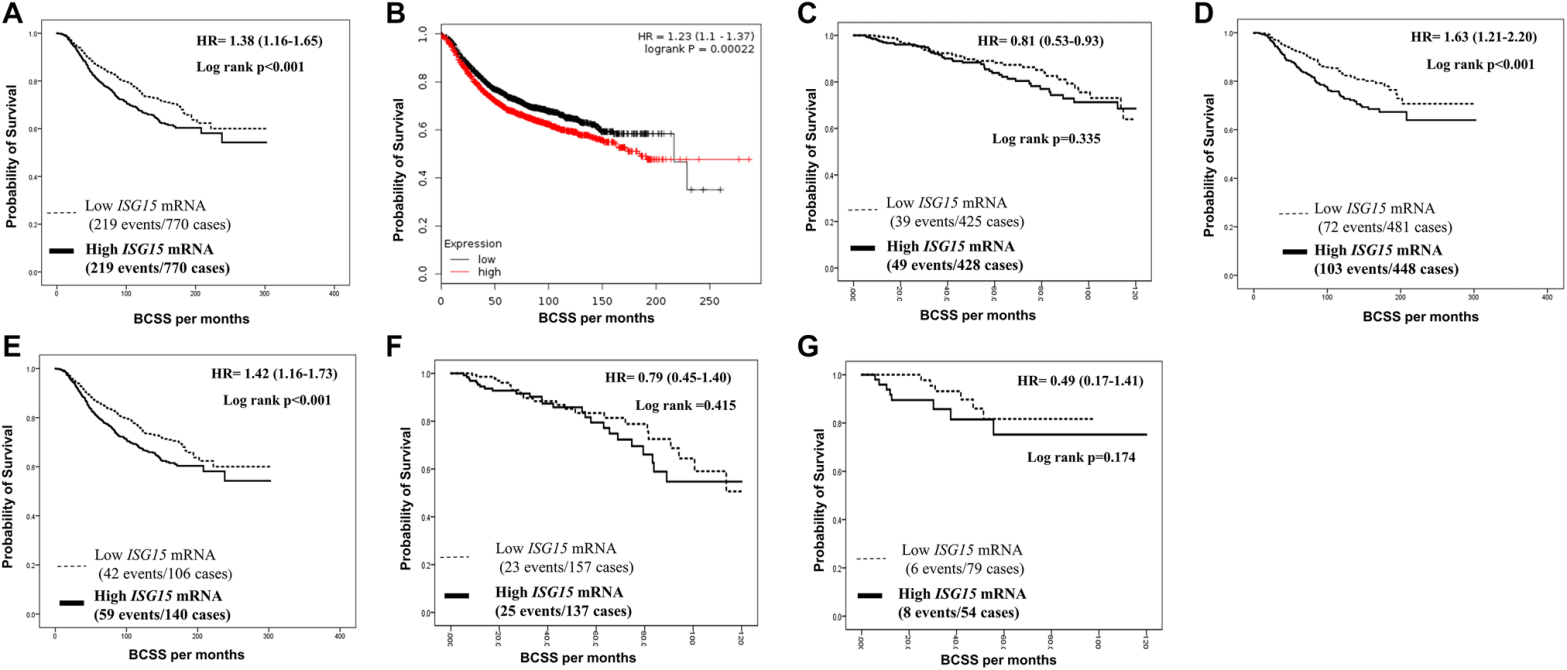
Patients’ outcomes of Breast cancer survival on Transcriptomic level. **a** Cumulative survival of BC patients stratified by *ISG15* mRNA expression in the METABRIC cohort. **b** Cumulative survival of BC patients stratified by *ISG15* mRNA expression in the KM-Plotter cohort. **c** Cumulative survival of BC patients *ISG15* mRNA expression in the TCGA cohort. **d** Cumulative survival of BC patients stratified by *ISG15* mRNA expression in the METABRIC-restricted LVI-positive cohort. **e** Cumulative survival of BC patients stratified by *ISG15* mRNA expression in the METABRIC-restricted HER2-positive cohort. **f** Cumulative survival of BC patients stratified by *ISG15* mRNA expression in the TCGA-restricted LVI-positive cohort. **g** Cumulative survival of BC patients stratified by *ISG15* mRNA expression in the TCGA-restricted HER2-positive cohort

In METABRIC and KM-Plotter dataset, outcome analysis indicated an association between high *ISG15* mRNA expression and shorter BCSS (*p* < 0.001, Fig. 1a and b). In TCGA, high ISG15 mRNA expression showed similar trend, but did not reach statistical significance (*p* = 0.335, Fig. 1c).

When we stratified the METABRIC mRNA cohorts based on LVI and HER2 status, high *ISG15* mRNA expression was associated with poor survival in LVI-positive subgroup (*p* < 0.001, Fig. 1d) and in the HER2-positive subgroup (*p* < 0.001; Fig. 1e). A trend towards similar associations with poor survival was observed in the TCGA cohort for both LVI-positive and HER2-positve cases that did not reach statistical significant (*p* = 0.415, Fig. 1f and *p* = 0.174, Fig. 1g, respectively). Nonetheless, when performing survival analysis to test the value of *ISG15* mRNA expression on both the METABRIC and TCGA cohorts after restriction to the LVI-negative and HER2-negative cases, our data showed no statistical association with patients outcome (*p* > 0.05) (Supplementary Fig. 2A, B, C and D, respectively).

### ISG15 pathway analysis

**Fig. 2:**
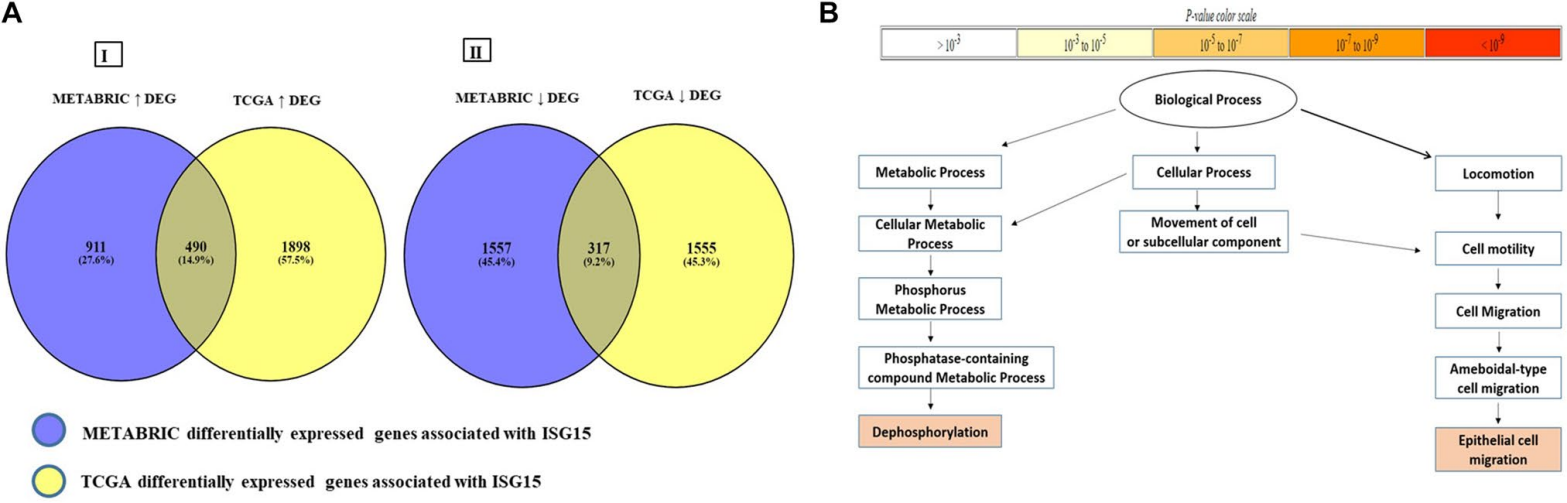
Differential gene expression and pathway analysis. **a** Overlapping differentially expressed genes associated with high ISG15 mRNA expression. Cases depicting (I) overexpressed differentially expressed genes associated with high ISG15 mRNA on both METABRIC and TCGA restricted on Luminal B and HER2-positive cohorts. (II) Overlapping downregulated differentially expressed genes associated with High ISG15 mRNA on both METABRIC and TCGA restricted on Luminal B and HER2-positive cohorts. DGE: differentially expressed gene, (↓): downregulated genes, (↑) overexpressed genes. **b** The enriched biological process generated by the common 490 overexpressed genes based on high ISG15 mRNA expression on both METABRIC and TCGA restricted on Luminal B and HER2-positive cohorts

Our DGE investigation identified a total of 807 differentially regulated genes associated with *ISG15* mRNA expression in both cohorts. Within the METABRIC cohort, high *ISG15 mRNA* expression displayed 1401 overexpressed and 1874 downregulated genes. Likewise, in the TCGA cohort, high *ISG15 mRNA* expression displayed 2389 overexpressed and 1872 downregulated genes. Remarkably, the overlapping of high and low DEGs of both cohorts between cases harbouring high *ISG15* mRNA expression against cases harbouring low *ISG15* mRNA expression in both cohorts included 490 common overexpressed and 317 common downregulated genes associated with *ISG15* mRNA expression (Fig. 2a). Analysis of the 490 commonly overexpressed genes identified over-represented gene ontology (GO) terms associated with epithelial cell migration which highlights the role of ISG15 in tumour oncogenesis. The common low-expressed genes showed no GO terms with enrichment *p* value above the specified *p* value threshold (*p* = 0.001) (Fig. 2b) (Supplementary Table 2).

### ISG15 protein expression

The expression of ISG15 showed significant correlation between transcriptomic level and protein level using Pearson correlation test (*p* = 0.042). Within the 14 BC full-face sections, normal breast terminal duct lobular unit (TDLU) showed faint to weak expression of ISG15 compared to higher expression of ISG15 in invasive breast tissue (Supplementary Fig. 1E and F, respectively). For dichotomisation into negative/low and high expression, the median H-score 35 was used. Out of 674 informative TMA cores, negative/low expression was observed in 275 cases (Supplementary Fig. 1G), while 399 cases (59%) showed high ISG15 expression (Supplementary Fig. 1H).

High expression of ISG15 protein was significantly associated with LVI positivity and other features of poor prognosis including younger age at diagnosis, larger tumour size, high grade, poor NPI, lack of expression of ER and PR and HER2 positivity (Table 3). Moreover, when we distributed the protein expression of ISG15 according to BC tumour IHC subtypes, high protein expression of ISG15 showed a significant association with ductal no special type (NST) BC tumour compared to lobular BC type (*p* = 0.003) (Table 3). Furthermore, when we compared ISG15 protein expression with BC progression-associated markers, high ISG15 protein expression was significantly associated with the high expression of P53, Ki67, EGFR and CD44, and high stromal immune markers of CD8, FOXP3 and CD68. However, it showed negative association with tumour E-cadherin expression (Table 3).

**Table 3:** Statistical association between ISG15 protein expression and clinicopathological characteristics of the studies cohort

Parameters	ISG15 protein expression		
	Low	High	*p* value
	*N* (%)	*N* (%)	
Tumour size			
≤ 2.0 cm	144 (45)	178 (55)	**0.044**
> 2.0 cm	127 (37)	216 (63)	
Nodal status			
Negative	393 (52)	369 (48)	0.192
Positive	165 (43)	219 (57)	
Histological grade			
1	43 (51)	42 (49)	**0.002**
2	104 (47)	117 (53)	
3	128 (35)	238 (65)	
Tumour histological subtypes			
Ductal (NST)	216 (39)	336 (71)	**0.003**
Lobular	43 (61)	28 (39)	
Medullary-like	11 (79)	3 (21)	
Special type	10 (37)	17 (63)	
Lymphovascular invasion			
Negative	164 (44)	210 (56)	** < 0.001**
Positive	62 (30)	144 (70)	
Nottingham prognostic index			
Good prognostic group	91 (51)	88 (49)	**0.005**
Moderate prognostic group	134 (38)	220 (62)	
Poor prognostic group	46 (35)	86 (65)	
Age			
≤ 50	92 (36)	163 (64)	**0.034**
> 50	179 (44)	232 (56)	
Oestrogen receptor			
Negative	49 (30)	133 (73)	** < 0.001**
Positive	222 (46)	263 (54)	
Progesterone receptor			
Negative	97 (34)	186 (66)	**0.008**
Positive	165 (45)	205 (55)	
Human epidermal growth factor receptor 2			
Negative	243 (44)	313 (56)	
			** < 0.001**
Positive	19 (21)	73 (79)	
P53			
Negative	201 (45)	250 (55)	**0.001**
Positive	58 (29)	141 (71)	
Ki67			
Negative	95(46)	111 (54)	**0.008**
Positive	120 (35)	226 (65)	
E-Cadherin			
Negative	109 (36)	268 (64)	**0.005**
Positive	109 (48)	120 (52)	
N-Cadherin			
Negative	52 (38)	84 (62)	0.904
Positive	151 (39)	238 (61)	
Basal-phenotype			
Negative	361 (79)	96 (21)	**0.010**
Positive	246 (71)	102 (29)	
Epithelial growth factor receptor			
Negative	222 (43)	298 (57)	**0.03**
Positive	43 (32)	90 (68)	
CD8			
Negative	116 (44)	147 (56)	**0.013**
Positive	84 (33)	167 (67)	
CD44			
Negative	76 (43)	100 (57)	**0.005**
Positive	44 (28)	72 (72)	
FOXP3			
Negative	86 (47)	99 (53)	**0.019**
Positive	125 (36)	222 (64)	
CD68			
Negative	85 (47)	96 (53)	**0.008**
Positive	114 (35)	212 (65)	
IHC subtypes			
Luminal A	100(45)	124 (55)	** < 0.001**
Luminal B	50 (33)	100 (67)	
Her2 enriched	26 (20)	103 (80)	
TNBC	19 (21)	73 (79)	

Significant correlations are in bold

**Fig. 3:**
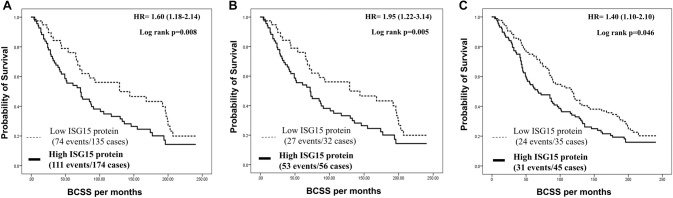
Patients’ outcomes of Breast cancer survival on Proteomic level. **a** Cumulative survival of BC patients stratified by ISG15 protein expression in the Nottingham cohort. **b** Cumulative survival of BC patients stratified by ISG15 protein expression in the Nottingham LVI-positive cohort. **c** Cumulative survival of BC patients stratified by ISG15 protein expression in the Nottingham HER2-positive cohort

Outcome analysis showed an inverse association between ISG15 protein expression and survival; high expression was associated with shorter BCSS (*p* = 0.008; Fig. 3a). Multivariate analysis demonstrated that high protein expression of ISG15 was associated with shorter BCSS [*p* = 0.026, Hazard ratio, 1.3, 95% CI 1.0–1.6], independent of other established prognostic factors including LVI, tumour size, histological grade, ER and HER2 status (Table 4).

**Table 4:** Multivariate Cox proportional hazard regression analysis for predictors of BCSS in the Nottingham BC cohort

Factors	BCSS		
	Hazard ratio	95% CI	*p* value
ISG15 expression	**1.27**	**1.03–1.56**	**0.026**
Tumour size	**1.70**	**1.37–2.12**	** < 0.001**
Tumour grade	1.25	1.00–1.57	0.066
Lymphovascular invasion	**1.85**	**1.50–2.23**	** < 0.001**
ER status	**0.675**	**0.53–0.861**	**0.002**
HER2 status	**1.73**	**1.33–2.25**	** < 0.001**

Significant correlations are in bold

When we stratified the Nottingham BC IHC cohort based on the LVI and HER2 status, high protein expression of ISG15 was associated with poor BCSS in the LVI-positive survival cases (*p* = 0.005, Fig. 3b), and in the HER2-positive survival cases (*p* = 0.046; Fig. 3c) subgroups, similar of poor survival trend were observed for LVI-negative survival cases and HER2-negative survival cases, but it did not reach statistical significant (*p* > 0.05, Supplementary Fig. 2E and F, respectively).

## Discussion

The differences in the morphological and molecular features of BC that determine behaviour, outcome and response to therapy are key characteristics that pose challenges in the management of BC patients. One of these challenges is the role of LVI in BC and understanding the underlying molecular mechanisms and drivers of LVI as potential therapeutic targets [1]. LVI is an essential process in the metastatic cascade that needs further studies. ISG15 expression is upregulated in various cancers, including breast [43], hepatocellular [44], lung [45], prostate [46] and bladder [47] cancers. However, the association between ISG15 and BC progression, particularly its role in LVI, yet remains to be defined. Our study indicates a significant correlation between high *ISG15* levels and not only LVI but also with other features of aggressive tumour behaviour including high tumour grade, large tumour size, hormone receptor negativity and overexpression of HER2, the immune cell markers, MMPs and the BC stem cell markers in addition to poor patient outcome. The association between high expression of *ISG15* and nodal status in the METABRIC cohort also supports its ability to contribute in BC invasion and metastasis. These results are in accordance with several previous studies, which demonstrated that ISG15 is significantly associated with cancer progression [17, 43–46]. Although in our study some variation in the association between ISG15 expression and clinicopathological variables such as nodal status, this can be attributed to the difference in the nature of cohort, convoluted post-transcriptional mechanisms or perhaps due to the substantial differences in in vivo half-lives of proteins [48, 49].

As observed in our study, elevated ISG15 expression is an independent factor of HER2 expression indicating that targeting ISG15 may be an attractive treatment option for HER2-positive and/or hormone receptor-negative tumour which is accompanied with severe type of BC that has no target therapy yet as they mainly showed resistance to hormonal pathway drugs [50, 51]. In mouse BC models, the presence of ISG15 showed a significant impact in therapeutic experiments via controlling CD8 expression in both primary and metastatic burden [44]. In the current study, there is a high immune response illustrated by high expression of CD8, FOXP3 and CD68, which play an important role in tumour microenvironment and immune response, to ISG15 protein expression which may also support the role of ISG15 in controlling tumour microenvironment in BC. It is well known that the tumour microenvironment has an essential role in stromal infiltration, invasion and development of LVI [52]. Additionally, *ISG15* mRNA association with MMPs indicates the role in tumour progression as they have the ability to reorganise the extracellular matrix and prepare the microenvironment to produce cytokines that enhance cancer cell migration and proliferation [53]. Our data are in accordance with the previous studies for ISG15 in BC which indicated that ISG15 is significantly associated with cancer development and metastasis [8, 54].

When investigating the correlation between *ISG15* mRNA expression and well-established EMT transcription factors, the results revealed a negative correlation with *CTNNB, CDH1;* however, a positive association was shown with *LLGL2*. These findings indicate that high ISG15, at both transcriptomic and proteomic levels, involves in promoting tumour cell migration and enhancing the LVI process. This may occur by activating the ISGlyation that inhibits cancer cell-stabilising proteins through F-actin activation, which might also correlate with inducing proliferative proteins such as P53, as our data allude [45, 55]. Furthermore, during metastasis, high levels of ISG15 might enhance a cooperative signalling by employing fibronectin-binding integrins such as αVβ3 and/or α5β1 to maintain high ISG15 levels. These integrins might trigger GTPases is resulting in activation or polymerisation of F-actin networks and stress fibres, which are necessary to stimulate the cell migration elements such as membrane protrusions, cell contractility and adhesion enforcement [8]. *CTNNB1* plays an important role in tumour cells adhesion and maintains them together via controlling the cell growth and adhesion between cells [56]. Prompting of *LLGL2* plays a key role in EMT activation and was positively associated with high *ISG15* mRNA expression. In addition, we have previously reported that downregulation of E-cadherin (*CDH1),* which was negatively correlated with high protein expression of ISG15, has an important role in EMT activation, migration and invasion in BC by regulating Wnt and PI3K signalling [38]. Moreover, elevated levels of N-cadherin (CDH2), which increases the production of MMP9 to initiate the ideal environment for migratory tumour cells by rupturing the basement membrane in the tumour primary site, would eventually promote the migration process [57]. However, the last scenario might not necessarily be speculated in our study because no significant association was detected between ISG15 levels and CDH2. This study presented that high ISG15 at the transcriptomic (METABRIC cohort) and proteomic level was associated with EGFR, which can control EMT, migration and invasion [58]. Collectively speaking, the increased levels of LLGL2, EGFR and decreased level in CDH1, recorded in the current study, promote EMT and cancer cell migration in BC [9]. Hence, the association between ISG15 and loss of CDH1 may result in a reciprocal inhibitory mechanism promoting cell migration and invasion, which may imply an indirect role of ISG15 in LVI.

In the whole BC cohort, high expression of ISG15 protein was an independent prognostic marker for worse BCSS. Among defined BC molecular subgroups, high ISG15 protein expression was observed to have the lowest survival rate in HER2-enriched BC. HER2 BC type was considered as a model in this study because it is an aggressive cancer that strongly associated with cancer cell migration and metastasis. One of the LVI prerequisites is the presence of specialised protrusions namely filopodia or lamellipodia to degrade the basement membrane through polymerisation of F-actin chain [59]. Therefore, targeting candidate biomarkers that modulate F-actin in cancer cells may help tailoring the treatment strategy [60–62]. It was reported that ISG15 pathway induces BC cell conformational change leading to increase the tumour cell motility and metastatic ability through F-actin and microtubule filament modulation [9]. In this study, the data of DEGs and pathway analysis have supported the proposed role of high ISG15 association with epithelial cell migration particularly for Her2 and luminal B subtypes. This could explain the reflection of poor prognosis and tumour development when ISG15 is highly expressed. Therefore, targeting ISG15 pathway may potentially help to find new avenues that can be useful to precisely inhibit the tumour progression using personalised medicines. Therefore, further gene ontology analyses to other IHC subtypes are recommended to better understand the ISG15 role in all BC subtypes.

The crosstalk between tumour cells and tumour microenvironment is well known as a complex process. Several of non-tumour cell factors contribute to this process and may play a critical role by inducing tumour aggressiveness and tumour cells' migration ability into the lymphatic vessels and cause LVI. Therefore, further mechanistic studies are warranted to understand the underlying mechanisms of LVI. Furthermore, ISG15 protein might be expressed in other cells; however, this is beyond the scope of this study which focuses mainly on the expression in tumour epithelial cells. Likewise, appropriate assessment for other type of cells in TMA is challenging as our TMAs were constructed primarily to incorporate tumour epithelial cells so the microenvironment may not be wholly representative.

In conclusion, our findings provide evidence that increased ISG15 at the transcriptomic and proteomic levels is strongly associated with LVI positivity and poor patient outcome in BC. Further functional in vivo and in vitro studies are warranted to identify its underlying mechanistic role and its therapeutic potential.

## Electronic supplementary material

Below is the link to the electronic supplementary material.

Supplementary file1 (JPG 186 kb)

Supplementary file2 (JPG 2381 kb)

Supplementary file3 (DOCX 23 kb)

Supplementary file4 (DOCX 22 kb)

## Data Availability

The authors confirm the data that have been used in this work are available on reasonable request.
