# Supplementary figures and images for: The prognostic significance of interferon-stimulated gene 15 (ISG15) in invasive breast cancer
Source: Breast Cancer Res Treat. 2020 Oct 19;185(2):293–305. doi: 10.1007/s10549-020-05955-1 (PMC7867506; doi:10.1007/s10549-020-05955-1)

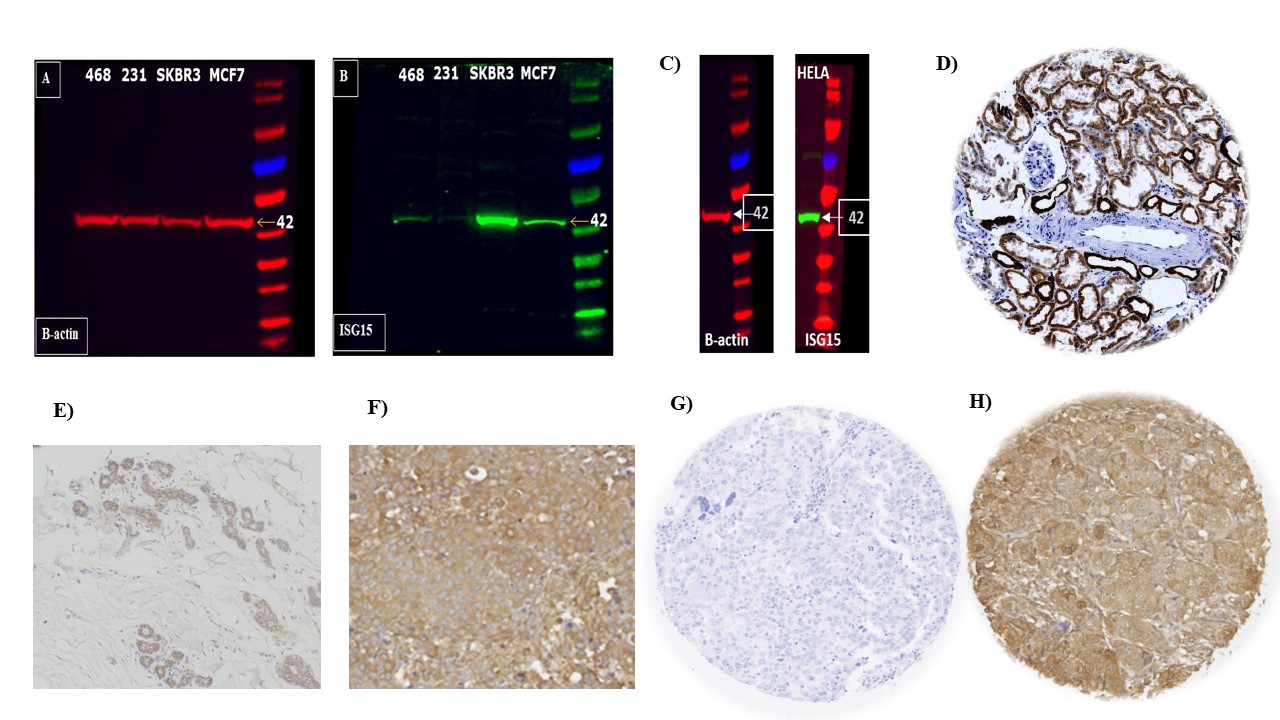

Supplement: Supplementary file 1 — Supplementary file1 (JPG 186 kb) [file 10549_2020_5955_MOESM1_ESM.jpg]

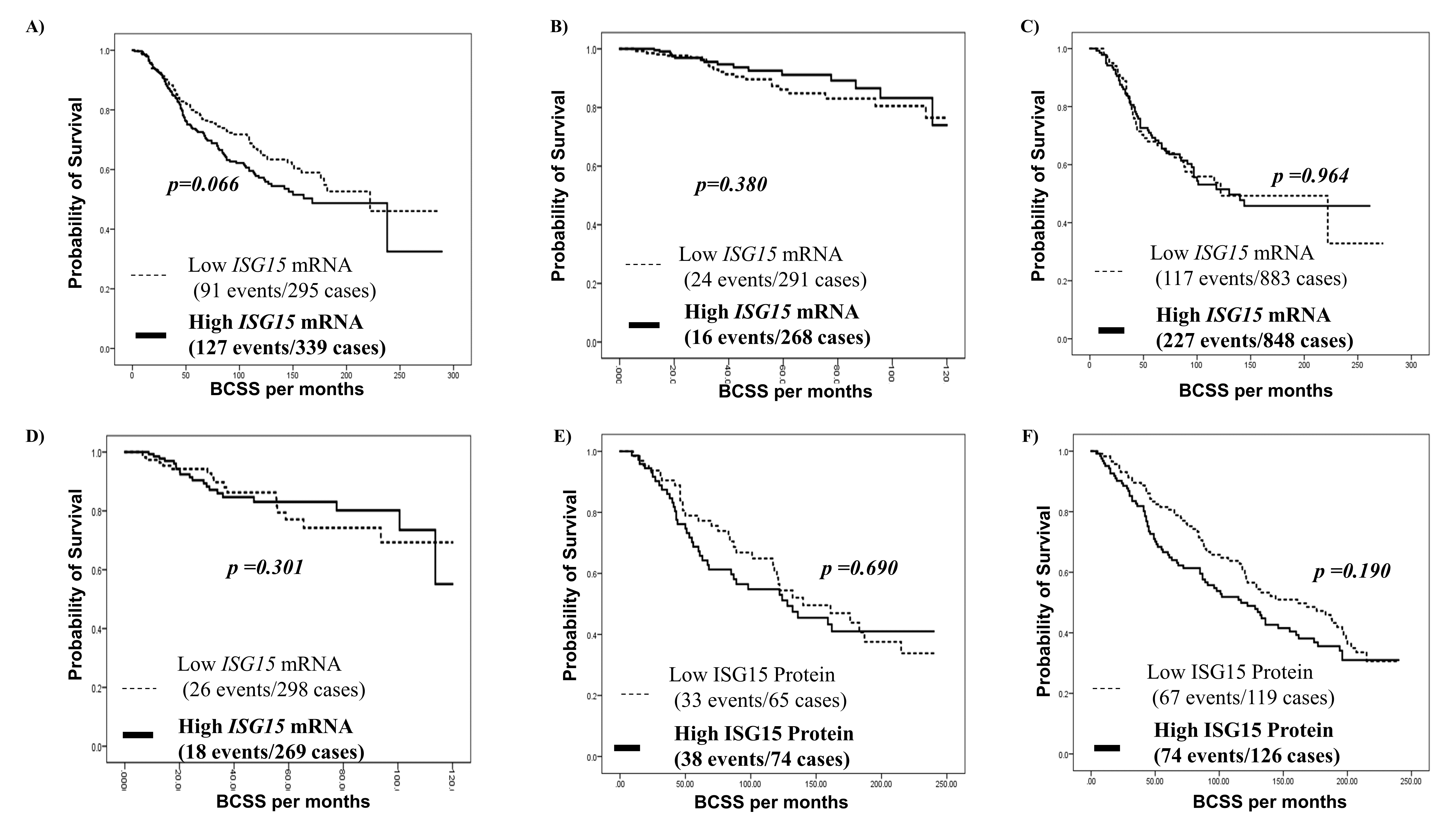

Supplement: Supplementary file 2 — Supplementary file2 (JPG 2381 kb) [file 10549_2020_5955_MOESM2_ESM.jpg]
