# Supplementary material for: The prognostic significance of interferon-stimulated gene 15 (ISG15) in invasive breast cancer
Source: Breast Cancer Res Treat. 2020 Oct 19;185(2):293–305. doi: 10.1007/s10549-020-05955-1 (PMC7867506; doi:10.1007/s10549-020-05955-1)
Supplement: Supplementary file 3 — Supplementary file3 (DOCX 23 kb) [file 10549_2020_5955_MOESM3_ESM.docx]

**Supplementary Table 1:** Association of ISG15 protein expression and pervious protein data results from our cohort.

| Proteins/Markers | Location of expression | Cut-off point | Reference |
| --- | --- | --- | --- |
| P53 | Nucleus of:   - Special‐type invasive breast carcinoma (including tubular, mucinous, medullary and papillary types). - Classic invasive lobular carcinoma. - Infiltrating ductal invasive breast carcinoma (including alveolar, solid and pleomorphic types). - Other miscellaneous tumours. | (>10% for P53 as high expression). | (30) |
| Ki67 | Cytoplasm of primary invasive lobular breast cancer. | (>20% for Ki67 as high expression). | (29) |
| EGFR | Cytoplasm of primary invasive lobular breast cancer. | (H-score >10 as high expression). | (35) |
| E-cadherin | Nuclear membrane of invasive breast cancer. | (H-score >100 as high expression). | (31) |
| CD44 | Cytoplasm of invasive breast cancer. | H-score >50 as high expression). | (32) |
| CD8 | Stromal Cytotoxic T infiltrative Lymphocyte. | (>1 cells as high expression). | (33) |
| FOXP3 | Nuclear of stromal T-regulatory infiltrative Lymphocyte. | (>1 cells as high expression). | (33) |
| CD68 | Stromal infiltrative Macrophage. | (>17 cells as high expression). | (34) |
