# Supplementary material for: The prognostic significance of interferon-stimulated gene 15 (ISG15) in invasive breast cancer
Source: Breast Cancer Res Treat. 2020 Oct 19;185(2):293–305. doi: 10.1007/s10549-020-05955-1 (PMC7867506; doi:10.1007/s10549-020-05955-1)
Supplement: Supplementary file 4 — Supplementary file4 (DOCX 22 kb) [file 10549_2020_5955_MOESM4_ESM.docx]

| **GO term** | **Description** | [P-value](http://cbl-gorilla.cs.technion.ac.il/GOrilla/lnmcjipu/GOResults.html#p_value_info) | [FDR q-value](http://cbl-gorilla.cs.technion.ac.il/GOrilla/lnmcjipu/GOResults.html#fdr_info) | [Enrichment (N, B, n, b)](http://cbl-gorilla.cs.technion.ac.il/GOrilla/lnmcjipu/GOResults.html#enrich_info) | [Genes](http://cbl-gorilla.cs.technion.ac.il/GOrilla/lnmcjipu/GOResults.html#genes_info) |
| --- | --- | --- | --- | --- | --- |
| [GO:0010631](http://www.godatabase.org/cgi-bin/amigo/go.cgi?query=GO:0010631&view=details) | epithelial cell migration | 1.33E-04 | 6.34E-01 | 79.17 (475,2,6,2) |  |
|  |  |  |  |  | PKN1 - protein kinase n1 |
|  |  |  |  |  | PTP4A3 - protein tyrosine phosphatase type iva, member 3 |
| [GO:0016311](http://www.godatabase.org/cgi-bin/amigo/go.cgi?query=GO:0016311&view=details) | dephosphorylation | 3.03E-04 | 7.21E-01 | 95.00 (475,5,2,2) |  |
|  |  |  |  |  | PTP4A3 - protein tyrosine phosphatase type iva, member 3 |
|  |  |  |  |  | TMEM55A - transmembrane protein 55a |

**Supplementary Table 2:** Biological process analysis using common differentially overexpressed genes associated with high ISG15 expression.

**'P-value'** is the enrichment p-value computed according to the mHG or HG model. This p-value is not corrected for multiple testing of 4755 GO terms.

**'FDR q-value'** is the correction of the above p-value for multiple testing using the Benjamini and Hochberg (1995) method.
Namely, for the i^th^ term (ranked according to p-value) the FDR q-value is (p-value * number of GO terms) / i.

**Enrichment (N, B, n, b)** is defined as follows:
N - is the total number of genes
B - is the total number of genes associated with a specific GO term
n - is the number of genes in the top of the user's input list or in the target set when appropriate
b - is the number of genes in the intersection
Enrichment = (b/n) / (B/N)
